# Genetic disorders of thyroid metabolism and brain development

**DOI:** 10.1111/dmcn.12445

**Published:** 2014-03-26

**Authors:** Manju A Kurian, Heinz Jungbluth

**Affiliations:** 1 Developmental Neurosciences, UCL-Institute of Child HealthLondon, UK; 2 Department of Neurology, Great Ormond Street Hospital for ChildrenLondon, UK; 3 Department of Paediatric Neurology, Evelina Children's Hospital, Guy's & St Thomas' NHS Foundation TrustLondon, UK; 4 Randall Division for Cell and Molecular Biophysics, Muscle Signalling Section, King's College LondonLondon, UK; 5 Clinical Neuroscience Division, Institute of Psychiatry, King's College LondonLondon, UK

## Abstract

Normal thyroid metabolism is essential for human development, including the formation and functioning of the central and peripheral nervous system. Disorders of thyroid metabolism are increasingly recognized within the spectrum of paediatric neurological disorders. Both hypothyroid and hyperthyroid disease states (resulting from genetic and acquired aetiologies) can lead to characteristic neurological syndromes, with cognitive delay, extrapyramidal movement disorders, neuropsychiatric symptoms, and neuromuscular manifestations. In this review, the neurological manifestations of genetic disorders of thyroid metabolism are outlined, with particular focus on Allan-Herndon-Dudley syndrome and benign hereditary chorea. We report in detail the clinical features, major neurological and neuropsychiatric manifestations, molecular genetic findings, disease mechanisms, and therapeutic strategies for these emerging genetic ‘brain-thyroid’ disorders.

## What this paper adds

- An overview of the neurological manifestations of thyroid dysfunction.
- Detailed characterization of the genetic ‘brain-thyroid’ disorders.

The thyroid gland plays a fundamental role in normal human development and maintenance, and it is therefore not surprising that genetic and acquired disorders of thyroid metabolism often include prominent neurological dysfunction (TableI). The classical example of congenital hypothyroidism (or ‘cretinism’^1^) clearly demonstrates this association between abnormal thyroid function and neurological deficits. Affected infants are at substantial risk of neurocognitive difficulties if l-thyroxine treatment is delayed, whereas prompt institution of treatment usually results in normal intellectual development.^2^ Other neurological features seen in congenital and later onset hypothyroidism include anxiety, depression, carpal tunnel syndrome, peripheral neuropathy, headache, visual field defects, and coma. At the other end of the spectrum, patients with hyperthyroidism have been reported to have an even wider range of neurological symptoms, including anxiety, depression, psychosis, encephalopathy, marked tremor, Graves ophthalmopathy, muscle weakness, and even cerebrovascular events.^3^,^4^ Less frequently, deterioration in cognitive function (affecting memory, attention, and planning), headaches, seizures, myasthenia, dysphonia, paroxysmal dyskinesias, and chorea^5^,^6^ have been found in association with hyperthyroid states.

**Table I:** Aetiology, and biochemical and clinical features of hypothyroidism, hyperthyroidism, Allan-Herndon-Dudley syndrome, and benign hereditary chorea

	Hypothyroidism	Hyperthyroidism	AHDS	BHC
Aetiology				
	Multiple aetiologies including:	Grave's Disease (GD)	X-linked Mutation in *SLC16A2*	Autosomal dominant Mutation in *NKX2.1*
	Congenital hypothyroidism			
	Pituitary failure (PF)			
	Autoimmune thyroiditis (AT)			
	Hashimoto's encephalopathy (HE)			
	Myxoedema coma			
Biochemical features				
TSH	↑ (↓in PF)	↓	N/mild ↑	↑
Free T4 (thyroxine)	↓	↑	N/mild ↓	↓
Free T3 (triiodothyronine)	↓	↑	↑↑ (↓reverse T3)	↓
Antibodies (Ab)	Thyroid peroxidase, thyroglobulin, thyroid microsomal Ab (AT, HE)	TSH receptor stimulating Ab (GD)	−	−
Neurological features				
Chorea	−	++	++	+++
Dystonia	−	+	++	++
Paroxysmal dyskinesia	−	+	++	+
Cognitive impairment	++ (congenital subgroup)	+	+++	+
Depression	++	++	−	+
Anxiety	++	+++	−	−
Seizures	−	+	+	−
Hypotonia	++ (congenital subgroup)	−	++	++
Muscle weakness	++	++	++	++
Myasthenia gravis	+	+	−	−
Periodic paralysis	−	+	−	−
Carpal tunnel	+	+	−	−
Tremor	−	+++	+	+
Cerebellar ataxia	+	−	+	+
Peripheral neuropathy	+	+	−	−
Stroke	+	+	−	−
Headache	+	+	−	−
Dysphonia	+	+	−	−

AHDS, Allan-Herndon-Dudley syndrome; AT, autoimmune thyroiditis; BHC, benign hereditary chorea; GD, Graves disease; HE, Hashimoto encephalopathy; N, normal; PF, pituitary failure; T_3_, triiodothyronine; T_4_, thyroxine; TSH, thyroid-stimulating hormone; (−), Clinical Feature not usually reported; (+), Clinical Feature sometimes reported; (++), Clinical Feature commonly reported; (+++), Clinical Feature reported in most cases.

The pathophysiological processes underpinning the neurological symptoms in hypothyroid and hyperthyroid states are likely to be multifactorial, and include (1) autoimmune mechanisms, for example in Hashimoto encephalopathy (thyroid peroxidase, thyroglobulin, or thyroid microsomal thyroid autoantibodies, association with autoimmune vasculitis), Graves disease (thyroid-stimulating hormone receptor antibodies), and thyroid dysfunction related to myasthenia gravis; (2) ‘channelopathy’ as seen in thyrotoxic periodic paralysis; (3) adrenergic hypersensitivity associated with hyperthyroid tremor; and (4) ischaemia in vascular strokes in patients with hyperthyroid and atrial fibrillation.^4^ However, the primary processes governing many neurological features (e.g. cognitive dysfunction and psychiatric symptoms) in abnormal thyroid states are not yet fully elucidated, and are likely to be complex as a consequence of both the primary and secondary effects of thyroid dysfunction on metabolic cellular processes and neuronal networks.

The neurological spectrum associated with hypothyroid and hyperthyroid states has rarely been systematically reviewed in the literature,^4^ particularly with regard to childhood manifestations. In addition to inborn errors of thyroid metabolism and acquired thyroid disorders, more recently, distinct genetic entities affecting normal formation and function of both brain and thyroid have been recognized. Although often associated with a wider range of neurological manifestations, movement disorder phenotypes feature prominently in these conditions. This is particularly interesting given the association of chorea and dyskinesia with hyperthyroid states, suggesting a euthyroid state may have a role in normal control of movement. In this review we focus on the clinical features, molecular genetic findings, pathophysiological disease mechanisms, and therapeutic strategies for this emerging group of genetic ‘brain-thyroid’ disorders.

## Allan-Herndon-Dudley Syndrome

In 1944, William Allan, Nash Herndon, and Florence Dudley reported a large North American pedigree spanning six generations with 24 affected males characterized by a distinct combination of dysmorphic features, intellectual disability, and associated neurological findings.^7^ Further familial male cases with similar clinical features and additional characteristic thyroid hormone abnormalities were subsequently reported,^8^–^14^ resulting in the recognition of Allan-Herndon-Dudley syndrome (AHDS; OMIM 300523) as a distinct X-linked intellectual disability syndrome. The subsequent identification of mutations in the *SLC16A2* gene encoding the monocarboxylate transporter 8 (MCT8)^9^,^15^ established AHDS as the first genetically resolved neurodevelopmental disorder due to defective thyroid hormone metabolism.

### Disease onset

Presentation is typically from birth or early infancy. Suggestive thyroid abnormalities are usually detectable early in life, whereas dysmorphic features and in particular neurological symptoms evolve over time.

### Neurological/neuropsychiatric features

**Figure 1:**
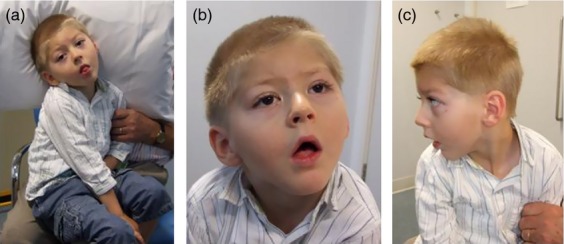
A 4-year-old patient with genetically confirmed Allan-Herndon-Dudley syndrome, with photographs illustrating (a) face/trunk/limbs (b) facial features and (c) lateral facial view. Note the typical but subtle dysmorphic features, including myopathic facial appearance with inverted V-shaped mouth, low anterior hairline, narrow forehead, receding chin, and large, low-set ears. From Gika et al.,^17^ with permission.

Neurological features associated with AHDS are variable but evolve along a predictable trajectory throughout development, with profound hypotonia prominent in early infancy and gradual evolution of a spastic paraplegia throughout childhood.^10^ Profound learning difficulties and global developmental delay are common and most affected children cannot walk or talk. A recognizable, particularly placid and sociable personality trait has been suggested. A dysmorphic mainly myopathic facial appearance (Fig.1), muscle wasting, contractures, pectus excavatum, and a scoliosis are often associated. There may be an associated rotatory nystagmus and dysconjugate eye movements.^9^ A complex movement disorder usually evolves over time^16^,^17^ and may comprise dystonic posturing and choreoathetosis. Paroxysmal dyskinesias, occurring spontaneously or triggered by external stimuli such as sudden positional changes, are a peculiar and often particularly distressing feature^17^,^18^ that may be difficult to distinguish from ‘seizures’ reported to be present in a few individuals.^19^

### Systemic disease features

General features associated with AHDS^10^,^20^ are variable, but often include a dysmorphic, elongated facial appearance with ptosis, bitemporal narrowing and simplified ears (Fig.1), and a head circumference at the lower end of the normal range (0.4–9th centile). The diagnosis may be easily missed, as evidenced by a substantial proportion of adults with genetically unexplained intellectual disability in whom *SLC16A2* mutations were retrospectively identified.^21^ Many affected males may have tachycardia and difficulties gaining weight, reflecting a degree of peripheral hyperthyroidism in tissues where thyroid hormone transport is not MCT8-dependent.

### Differential diagnosis

Particularly in cases where dysmorphic facial features are not suggestive, a syndromic diagnosis of AHDS is often not immediately suspected. The differential diagnosis evolves with the typical clinical features over time, and may include primary neuromuscular conditions, dystonic/dyskinetic forms of cerebral palsy, and other causes of X-linked intellectual disability. Of note, absent or reduced myelination in the context of an evolving spastic paraplegia in males with AHDS may lead to the misdiagnosis of Pelizaeus-Merzbacher disease,^17^,^22^ a disorder of myelination due to mutations in the *PLP1* gene on Xq22. *SLC16A2* mutation screening has therefore to be considered in males presenting with features of Pelizaeus-Merzbacher disease or X-linked spastic paraplegia,^23^ an allelic condition, once *PLP1* mutations have been excluded.

### Laboratory findings

A specific combination of thyroid hormone abnormalities reflective of increased renal thyroxine to triiodothyronine conversion and altered thyroid gland hormone secretion^15^,^20^,^22^,^24^ is the most distinctive laboratory marker of AHDS and should prompt *SLC16A2* sequencing in males with other indicative features. Thyroid hormone abnormalities suggestive of AHDS include normal or slightly elevated serum thyroid-stimulating hormone levels, normal or mildly decreased free thyroxine levels and, in particular, high free or total triiodothyronine levels with a reduced reverse high serum triiodothyronine concentration;^25^ as free, reverse, and total triiodothyronine are not always part of the routine laboratory thyroid assessment, the tests will have to be specifically requested if there is a high clinical suspicion of AHDS.^26^ A distinct thyroid follicular pathology has been reported in mtm^−/−^ knockout mice and ought to be anticipated in patients with AHDS.^27^ Thyroid function should be monitored in heterozygous female mutation carriers during pregnancy, as deterioration of thyroid function may occur and adversely affect even genetically unaffected fetuses.^28^

Other less specific laboratory abnormalities include increased serum lactate concentrations, probably reflective of a hyperthyroid metabolic myopathy,^29^ and increased serum sex hormone-binding globulin concentrations, probably reflecting a thyroid hormone effect on the liver. Increased ferritin and decreased cholesterol levels have been observed in some individuals.^30^

### Neuroimaging

A number of brain magnetic resonance imaging (MRI) studies have indicated abnormal white matter development in AHDS,^17^,^20^,^22^,^31^ supported by complementary magnetic resonance spectroscopy findings in selected cases.^17^,^32^ Where studied longitudinally, brain MRI in patients with AHDS shows absent or markedly reduced myelin at baseline that develops slowly over time^17^,^22^ but remains substantially delayed on follow-up. The brain MRI appearance is similar to that of Pelizaeus-Merzbacher disease.^17^,^22^ However, in contrast to *PLP1*-related Pelizaeus-Merzbacher disease, myelination appears to complete, eventually, in older AHDS patients.

Other inconsistent MRI abnormalities reported in some individuals with AHDS include subtle cortical, subcortical^25^ and cerebellar atrophy,^33^ as well as high T2 signal in the putamina.^34^,^35^

### Treatment

#### Movement disorder

Drugs usually given in the management of dystonic or choreoathetoid movement disorders may also be considered, but their efficacy has not been systematically evaluated in patients with AHDS.

#### Thyroid dysfunction

Although thyroid hormone replacement may cause improvement or normalization of laboratory markers, no convincing effect on the neurological phenotype has been observed.^36^,^37^

As the psychomotor retardation is thought to be caused by impaired neuronal triiodothyronine uptake during brain development, pharmacological efforts have concentrated on triiodothyronine analogues that do not require MCT8 for neuronal uptake.^24^ A recent pilot study with diiodothyropropionic acid,^30^ a thyroid hormone analogue that does not rely on MCT8 for tissue entry, resulted in normalization of laboratory thyroid parameters and reversed signs of peripheral hyperthyroidism but did not improve neurological features. The recent finding that the thyroid hormone analogue 3,5,3′,5′-tetraiodothyroacetic acid (tetraiodothyroacetic acid or tetrac) stimulates thyroid hormone neuronal differentiation in mct8^−/−^ mice suggests a novel pharmacological agent that may be of potential therapeutic benefit in patients with AHDS.^38^

### Molecular genetics and gene function

Causative mutations in the *SLC16A2* (solute carrier family 16, member 2) gene on Xq13.8 have now been identified in more than 170 males from 90 families and some heterozygote carrriers,^39^,^40^ and probably also accounts for a proportion of adult males with unresolved X-linked intellectual disability.^21^ *SLC16A2* belongs to the 14 members of the SLC16 family of genes^41^ and encodes the MCT8 protein. The majority of *SLC16A2* mutations results in complete loss of function, although few mutations with clinically milder phenotypes may show residual transporter activity. Known pathogenic *SLC16A2* missense mutations localize exclusively to the transmembrane helices within the MCT8 protein.^42^

MCT8 is an active and specific thyroid hormone transporter with differential tissue expression.^43^–^46^ In humans, MCT8 shows preferential substrate specificity for triiodothyronine and is early and widely expressed in the brain (where its function appears most critical) and other tissues including the heart, liver, kidneys, and skeletal muscle.^24^,^45^

In vitro studies have demonstrated that *SLC16A2* mutations result in a reduced or absent supply of triiodothyronine to neurons^47^ and that genotype-phenotype correlations largely reflect the residual triiodothyronine transport capacity of the mutant MCT8 transporter on the neuronal level,^48^ corresponding to the crucial role of thyroid hormones in brain development.^46^,^49^,^50^ More specifically, triiodothyronine induces differentiation of the oligodendrocyte precursor, acts as a survival factor for oligodendrocytes^51^ and affects the distribution of myelin proteins at later stages of oligodendroglial development.^52^ Although patients harbouring *SLC16A2* mutations very rarely exhibit signs of clinically overt hyperthyroidism,^29^ additional non-neurological symptoms are likely to reflect a relative hyperthyroid state in tissues that are not exclusively dependent on MCT8-mediated thyroid hormone uptake (e.g. the liver^25^ and muscle^33^).

Two animal models of MCT8 deficiency have been generated recently, the mct8^−/−^ mouse^53^ and a transgenic zebrafish.^54^ The mct8^−/−^ mouse accurately replicates the human thyroid profile,^55^,^56^ but does not show any of the neurological features seen in patients with AHDS, suggesting the presence of additional thyroid hormone transporters with a synergistic effect in the mouse brain. However, a recently generated transgenic zebrafish shows abnormalities of neuronal development in the brain and spinal cord,^54^ suggesting MCT8 as a crucial regulator during embryonic neuronal development.

## Benign Hereditary Chorea (Brain-Lung-Thyroid Disease)

First reported in 1967,^57^ benign hereditary chorea (BHC, OMIM 118700) is an autosomal dominant, childhood onset movement disorder characterized by non-progressive chorea. It is a rare disorder and approximately 30 disease-causing mutations have been reported in the literature to date.^58^ Over time, it has been recognized that in some patients, the neurological features are part of a multisystem disorder, where affected patients have additional thyroid and respiratory disease manifestations (brain-lung-thyroid syndrome or choreoathetosis, hypothyroidism, and neonatal respiratory distress, OMIM 610978). Isolated BHC and brain-lung thyroid syndrome are both caused by mutations in the thyroid transcription factor gene, *NKX2.1*.

### Disease onset

Benign hereditary chorea classically presents in childhood (median age 3y) and it rarely presents after adolescence.^59^ The majority of affected children have a history of delay in attaining early neurodevelopmental motor milestones^58^,^60^ and often present with delayed walking or with a ‘clumsy’ or ‘ataxic’ gait.^58^

### Neurological/neuropsychiatric features

Chorea is the predominant movement phenotype and is classically generalized, affecting the trunk and limbs. Specific triggers include premenstruation and pregnancy.^58^ The amplitude and severity of choreiform movements vary greatly in patients with a good long-term prognosis. In published series it appears that by early adulthood, the chorea seems to either stabilize or resolve^58^ Other extrapyramidal features, including limb/axial dystonia,^61^ ‘jerky’ dystonia,^62^ and myoclonus are also observed and can further contribute to delayed motor development and disability. Intention tremor,^57^ dysarthria,^61^ and gait disturbance^62^,^63^ are also reported. Comorbid psychiatric symptoms of depression,^64^ psychosis,^65^ motor vocal tics,^58^ and attention-deficit–hyperactivity disorder^58^ have also been reported in patients with BHC. In addition, a recent case series reported a single patient with adolescent onset obsessive-compulsive disorder.^60^ Cognitive dysfunction is also reported in patients with BHC^58^ and educational support may be needed for many children with BHC. Reasons for poor school performances are likely to be multifactorial^58^ and may include factors such as (1) the movement disorder affecting writing ability; (2) psychiatric comorbidities such as attention deficit hyperactivity disorder; (3) delayed treatment of hypothyroidism; and (4) perinatal hypoxic-ischaemic injury from respiratory distress.

### Systemic disease features

It has been reported that approximately 30 to 50% of all cases with *NKX2.1* mutations have the full triad of brain, lung, and thyroid involvement.^60^ Hypothyroidism is commonly reported in as many as two-thirds of patients with BHC.^58^ It manifests as either congenital hypothyroidism (elevated thyroid-stimulating hormone, low thyroxine) presenting in the neonatal period or as compensated hypothyroidism (elevated thyroid-stimulating hormone, normal thyroxine) detected later in childhood or even adulthood.^58^ Respiratory symptoms are less frequently reported (approximately half of BHC cases) and can include neonatal respiratory distress syndrome (alveolar syndrome due to surfactant deficiency), recurrent chest infections, asthma, and lung cancer.^58^ Additional clinical characteristics have been described in a number of cases with *NKX2.1* mutations. These include short stature with growth hormone deficiency^59^,^60^ webbed neck,^65^ microcephaly, facial dysmorphia,^59^ hypodontia,^66^ visual impairment,^60^ patent foramen ovale,^67^ and malabsorption,^59^ as well as pes cavus, kyphosis, duplex kidney, and lichen sclerosis. There is much variability between BHC cases, both with regards to the thyroid/lung involvement and the presence of other systemic features, and the reasons for this are currently not entirely clear. It is possible that the clinical phenotype may be influenced by the nature of the *NKX2.1* mutation, or that some patients with contiguous gene deletions may have additional clinical features attributed to other genes also involved in the copy number variant encompassing *NKX2.1*. In addition, other currently undetermined genetic and environmental factors may also play a role. As more genetically proven cases of BHC are reported in the literature, the phenotypic spectrum of the disease will become increasingly clear.

### Differential diagnosis

The combination of early onset gait abnormalities, chorea, and brain/lung involvement should trigger *NKX2.1* testing, but clinical diagnosis can be difficult in many cases. In the early stages of the disease, non-specific gait abnormalities may be similar to those seen in ‘dyskinetic’ or ‘ataxic’ cerebral palsy, as well as in acquired postinfectious and autoimmune chorea. Distinguishing BHC from *DYT11*-myoclonus-dystonia syndrome (due to mutations in the *SGCE* gene) can also be difficult.^60^ Asmus et al.^62^ attempted to identify clinical criteria differentiating BHC from genetically proven myoclonus-dystonia and concluded that early onset hypotonia, chorea in infancy, and possible association with thyroid or lung disease were good clues for differentiating BHC from this similar genetic movement disorder. In addition, neuropsychiatric disorders such as obsessive-compulsive disorder are much more commonly described in patients with *DYT11*-myoclonus-dystonia syndrome than *TITF1-*BHC.

### Laboratory findings

Patients with BHC may have thyroid function abnormalities detected on laboratory testing, including low thyroxine and high thyroid-stimulating hormone. As hypothyroidism is so readily treated with l-thyroxine, patients presenting with early onset chorea as well as other movement disorders and neurodevelopmental delay should have thyroid function tests as part of the initial diagnostic work-up. In addition, it is recommended that patients with mutation-positive BHC should have regular thyroid function tests to (1) monitor disease and thyroxine therapy in those with congenital hypothyroidism; and (2) monitor for the evolution of later onset thyroid disease in patients with BHC without congenital hypothyroidism, thereby allowing instigation of thryoxine replacement therapy in a timely manner.^58^

### Neuroimaging

Although neuroimaging studies have been normal in many patients with BHC,^60^ isolated structural abnormalities have been reported in a number of mutation-positive cases. These include microcephaly and persistent cavum septum pellucidum,^68^ agenesis of the corpus callosum,^59^ diencephalic cystic masses, and abnormal sella turcica.^69^ Volumetric analysis has shown a bilateral decrease in striatal volume.^70^ Abnormalities on functional imaging in patients with BHC have included hypometabolism of the basal ganglia and cortex with 18-fluoro-2-deoxy-glucose positron emission tomography^71^ and reduced striatal and thalamic uptake of technetium-99m-ethylcysteinate dimer.^72^

### Treatment

#### Movement disorder

Although the symptoms of chorea have been targeted with a wide variety of therapeutic strategies, the majority have proven to be largely ineffective. Previous studies have reported symptomatic improvement with (high-dose) levodopa.^73^,^74^ Gras et al.^58^ reported the beneficial effect of low-dose tetrabenazine (0.5mg/kg/d for children and 37.5mg/d in adults) for chorea and motor function that was sustained at mean follow-up of 1.5 years. Improvement of chorea with ropinirole treatment and partial response to propranolol has also been reported in a single case.^75^

#### Hypothyroidism

l-Thyroxine replacement therapy is advocated, with regular monitoring of thyroid function tests.

#### Respiratory symptoms

Symptomatic treatment is recommended with antibiotics for pulmonary infections and appropriate treatment for symptoms of asthma (e.g. bronchodilators and steroids).

### Molecular genetics and gene function

Linkage analysis^76^ and positional cloning identified *NKX2.1* as the causative gene^77^ for BHC and brain-lung-thyroid syndrome. *NKX2.1* (also known as *TITF1, TTF-1, TEBP*, or *NXK2A)* is located on chromosome 14q13. *NKX2.1* is a member of the NK gene family of highly conserved homeodomain-containing transcription factors, specifically encoding thyroid transcription factor 1 protein, which plays an important role in basal ganglia, thyroid, and lung organogenesis.^78^ To date, more than 30 different *NKX2.1* mutations have been identified,^58^ mainly de novo (two-thirds of cases) or inherited in an autosomal dominant fashion (one-third of cases)^58^ with reduced penetrance. Reported variants in BHC include whole gene deletions, as well as splice-site, frameshift, nonsense, and missense mutations. The majority of pathogenic mutations are postulated to either cause nonsense-mediated decay or cause truncation of the resultant protein before or within the DNA-binding homeobox domain (encoded by exon 3). Thus, loss-of-function *NKX2.1* mutations result in haploinsufficiency, and the resultant mutant protein has altered DNA-binding properties and is thereby unable to activate target genes.^79^ Murine models of disease were developed by Kimura et al.^80^ They found that the T/ebp^−/−^ homozygous state conferred lethality and that homozygotes were born dead, with a rudimentary bronchial tree, abnormal epithelium in their pleural cavities and absent lung parenchyma, no thyroid/pituitary gland, and extensive brain abnormalities, especially in the ventral forebrain. Furthermore, Sussel et al.^81^ have demonstrated that murine knockout homozygotes display abnormal neuronal migration from the pallidum to the striatum with subsequent depletion of both cholinergic and GABAergic neurons, further confirming the essential role of this gene in organogenesis of the thyroid, lung, and the central nervous system, in particular ventral forebrain and pituitary gland.

## Conclusion

Normal thyroid hormone metabolism is essential for the physiological function of a wide range of organ systems, including the brain. Inherited disorders affecting both normal brain and thyroid development and function (AHDS and BHC, the ‘brain-thyroid’ disorders) are rare but important to recognize, as they may mimic a wide range of neurological and neuropsychiatric presentations, including congenital neuromuscular disorders, cerebral palsy, primary movement disorders, certain leukodystrophies, and (X-linked) intellectual disability. Moreover, although none of these conditions can presently be cured, supportive interventions are available that may alleviate disease manifestations. More rational therapeutic approaches are currently still at the experimental stage but may benefit patients in the future.

## Abbreviations

AHDS: Allan-Herndon-Dudley syndrome
BHC: Benign hereditary chorea
MCT8: Monocarboxylate transporter 8
